# FSH priming and hormonal modulation of oocyte competence in *in vitro* maturation for infertility treatment: a systematic review and meta-analysis

**DOI:** 10.3389/fendo.2025.1682277

**Published:** 2025-10-17

**Authors:** Krystal Baysan Lin, Yungchen Chien, Jung-Hsiu Hou, Yen-Chen Wu, Ping Lun Lin, Li-Ting Chien, Chi-Huang Chen

**Affiliations:** ^1^ Division of Reproductive Medicine, Department of Obstetrics and Gynecology, Taipei Medical University Hospital, Taipei, Taiwan; ^2^ Graduate Institute of Medical Science, College of Medicine, Taipei Medical University, Taipei, Taiwan; ^3^ Graduate Institute of Biomedical Informatics, Taipei Medical University, Taipei, Taiwan; ^4^ Taipei Medical University Library, Taipei, Taiwan; ^5^ Department of Obstetrics and Gynecology, School of Medicine, College of Medicine, Taipei Medical University, Taipei, Taiwan

**Keywords:** gonadotropin, FSH, priming, IVM, ART

## Abstract

**Background:**

Does follicle-stimulating hormone priming improve reproductive outcomes in women undergoing *in vitro* maturation treatment for infertility?

Follicle-stimulating hormone (FSH) is a key endocrine regulator of oocyte folliculogenesis and is crucial for granulosa-oocyte communication and cytoplasmic maturation. *In vitro* maturation (IVM) offers a lower risk when compared with conventional *in vitro* fertilization ovarian stimulation; however, widespread clinical adoption is limited by variable success rates and protocol heterogeneity. In regard to optimization strategies, FSH priming has been proposed to enhance oocyte competence, but its impact remains debatable.

**Objective:**

To evaluate the effects of FSH priming on oocyte maturation and reproductive potential in IVM cycles for infertile women.

**Methods:**

Employing PRISMA guidelines, we systematically searched PubMed, Cochrane Library, Embase, and Web of Science for randomized controlled trials comparing FSH-primed versus non-primed IVM cycles in infertile women. The primary outcome was oocyte maturation rate, whereas secondary outcomes included fertilization rate, cleavage rate, pregnancy rate, and implantation rate. Data pooled used random-effects models, with heterogeneity assessed by *I^2^* statistic.

**Results:**

Six randomized controlled trials comprising of 497 women were analyzed. FSH priming was associated with a statistically significant increase in oocyte maturation rate [OR 1.24(95% CI, 1.05-1.45)] when compared with the non-stimulated group. However, pooled analysis showed no significant differences in fertilization rate or clinical pregnancy rate between groups.

**Conclusion:**

FSH priming has been shown to enhance oocyte maturation rate in *in vitro* maturation cycles. However, current evidence shows that gonadotropin does not significantly improve fertilization or pregnancy outcomes.

## Introduction

*In vitro* fertilization (IVF) revolutionized reproductive medicine in 1978, but the earliest attempts at embryo culture were actually based on *in vitro* maturation (IVM). Decades later, IVM is reclaiming its role as a clinically viable artificial reproductive technology (ART) option, especially for patients seeking alternatives to ovarian stimulation. Unlike standard IVF, which requires ovarian stimulation with gonadotropins, IVM minimizes hormonal exposure, reduces costs, and mitigates the risk of ovarian hyperstimulation syndrome (OHSS), an adverse outcome clinicians take active measures to prevent in women with polycystic ovarian syndrome (PCOS) and high antral follicle count (1, 2). Since the first live birth from IVM in 1991, clinical applications have expanded to include fertility preservation, poor responders, and cases where gonadotropin stimulation is contraindicated. Despite its advantages, IVM outcomes have historically lagged behind those of conventional IVF, with lower implantation and live birth rates. However, ongoing advancements in priming strategies, culture conditions, and embryo transfer protocols have revitalized interest in IVM as a viable treatment option. Reflecting this progress, the American Society for Reproductive Medicine (ASRM) Practice Committee announced in 2021 that IVM should not be considered an experimental procedure and recognized its potential for wider clinical implications (2, 3).

While IVM presents unique opportunities, its lower reproductive success rates remain a setback in the clinical scenario (4). What can be considered as a major factor influencing IVM outcomes is oocyte competence, since the process of nuclear maturation does not always align with cytoplasmic capacity for fertilization and embryo development. This has led to researchers to investigate various priming protocols in hopes of optimizing IVM protocols. FSH priming, hCG priming and dual priming approaches have been explored of capacity of improving oocyte quality, embryo formation, and implantation rates; however, findings remain inconsistent. Additionally, endometrial receptivity in IVM cycles differs from that in stimulated IVF cycles, raising much concern about synchronization between embryo development and the endometrial lining. Addressing these challenges is essential to improving IVM success rates and bringing about expanding this role in clinical practice (5).

One of the most debated aspects of IVM is the role of FSH priming, and whether or not this gonadotropin enhances oocyte maturation and further impacts the competence of embryo development. A previous study suggested that FSH priming enhances oocyte cytoplasmic and nuclear maturation, leading to higher maturation rate, pregnancy rates and implantation rates (6). However, others report no significant impact on clinical pregnancy outcomes, contributing to ongoing uncertainty regarding the benefits of FSH priming (7). Past studies have had large disparities in patient selection, stimulation protocols, and embryo transfer strategies, which could further complicate direct comparisons across studies. With much heterogeneity of existing research, a comprehensive analysis is needed to quantitatively synthesize evidence on the effects of FSH priming in IVM cycles.

The primary objective of our study is to determine whether FSH priming enhances IVM outcomes, particularly in terms of oocyte maturation, fertilization, implantation, and pregnancy rates. Additionally, we aim to assess whether FSH priming yields different results in PCOS versus normo-ovulatory patients, a subgroup that may have different outcomes because of altered follicular and hormone milieu, and thus offering insights into patient-specific responses. By consolidating available evidence, this study seeks to clarify the clinical utility of FSH priming, identify gaps in knowledge, and guide future research toward optimizing IVM protocols for improved reproductive success. As IVM technology evolves, well-designed randomized controlled trials and standardized methodologies will be essential in refining clinical guidelines and expanding its role in ART.

## Methods

### Search strategy

Our search of databases including PubMed, Cochrane, Embase, and Web of Science were executed on 28 August, 2024. A search strategy of free text terms was used for concepts of (1) *in vitro* maturation (2), immature oocytes (3), infertility, and (4) gonadotropin. Our search strategy followed the Preferred Reporting Items for Systematic Reviews and Meta-analysis (PRISMA) guidelines and adopted the following search terms: IVM (*in vitro* maturation) in combination with gonadotropin and randomized controlled trials. We also did additional screening with the reference lists of relevant literature to evaluate for potential eligible studies. The search strategy was limited to English. Citation searches were done manually to identify possible additional studies and relevant literature. Our last search date was on 26^th^ of March, 2025.

### Study selection

The types of studies included are randomized control trials done on infertility patients that were undergoing treatment with *in vitro* maturation. The patient population may include PCOS with infertility, tubal infertility, or male factor infertility that were to undergo *in vitro* maturation cycles for *in vitro* fertilization treatment. Comparison of intervention with FSH priming and a control group of non-stimulation would be required. Outcomes of MII maturation rate, cleavage rate, pregnancy rates were compared.

The search results were de-duplicated and carried out in EndNote21. After duplications were eliminated using Endnote’s identification strategy, the remaining studies were hand-searched for duplication. Then, these studies were screened by title and full text by YCW and KBL for studies with outcome measures of pregnancy and implantation rate for patients with infertility undergoing treatment for *in vitro* maturation for immature oocytes.

### Data extraction

Two reviewers (YCW and KBL) both independently evaluated the titles and abstracts of all studies that met inclusion criteria, and these studies were carefully assessed for relevance and eligibility. In cases of disagreement, a third author and final reviewer (JHH) was consulted to resolve and reach consensus. While seven studies were eligible for meta-analysis during data extraction, it was brought to attention that Mikkelsen 1999 and Mikkelsen 2000 had the exact same data and outcome for both the first experiments of studies that included FSH priming and no-FSH priming for comparison. After full review of the two studies, all three reviewers (KBL, YCW, and JHH) came to the conclusion to remove Mikkelsen 1999 from the meta-analysis, to prevent unintentional duplication of data, which could lead to an overestimation of outcomes.

Full texts of included studies were examined, and data collection of study characteristics, designs, intervention details, and primary/secondary outcomes were extracted for this study.

### Risk of bias regarding included studies (appraisal of methodological quality)

For risk of bias, the Cochrane Risk of Bias Tool for randomized controlled trials 2.0 (Rob 2.0) was utilized, and two reviewers (YCW and KBL) independently performed the evaluation. Evaluation with Rob 2.0 was performed across following domains: randomization process, deviation to intended interventions, missing outcome data, the measurement of outcomes, and selection of reported results (8). Risks of bias was classified into high, low, or some concern risk of bias regarding each domain.

### Statistical analysis

Data aggregation and statistical analysis were carried out under the PRISMA guideline. Meta-analyses were performed using Review Manager Web. Odds ratio (OR) with a 95% corresponding interval was used for dichotomous variables. A random-effects model was chosen to incorporate heterogeneity of studies. Heterogeneity between results of different studies was accounted for using I^2^ statistics, and classifying 25%-50% as low, 50%-75% as moderate, and >75% as high. The results were exhibited as forest plots, with outcomes of p-values < 0.05 considered as statistically significant. Subgroups of PCOS were performed specifically to take into consideration the population difference.

## Results

### Search results

**Figure 1:**
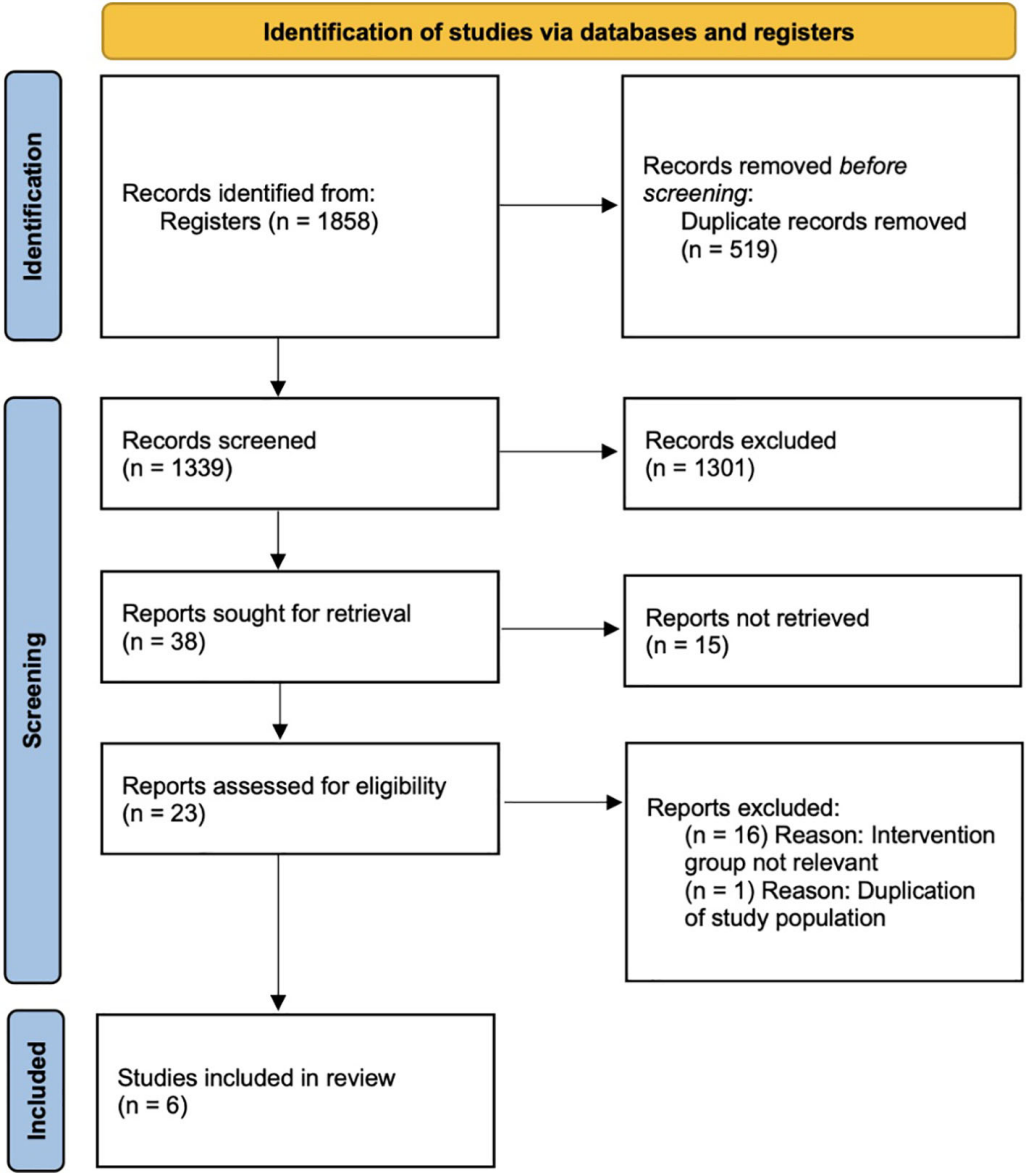
Prisma flowchart.

In our search, 1,858 records were identified initially for further screening. After deduplication and screening of titles and abstracts, 1,301 of the 1,339 deduplicated records were excluded because of irrelevance. The remaining 38 records were assessed for eligibility, and 15 were excluded because the topic was on IVM culture medium, and another 16 were excluded because the intervention group was focused on HCG priming and not FSH priming, which did not meet our inclusion criteria. One other study was removed to avoid duplication of data because of suspected same population. The remaining six RCTs were compatible with our eligibility criteria and were included into this systematic review. A flowchart depicting the search and selection process is presented in **Figure 1**.

### Characteristics of included studies

The six RCTs included in the meta-analysis comprise 497 participants, with 255 participants in the FSH treatment group and 242 participants in the unstimulated control group, and with a total of 2,898 oocytes in the FSH treatment and 2,947 oocytes in the unstimulated control group. The characteristics and outcomes of the studies are condensed in **Table 1**. All six studies were randomized controlled studies. Four studies included PCOS infertility patients, whereas the other two studies did not include POCS patients and only normo-ovulatory infertile patients with tubal or male factors (6, 9–11). All RCTs reported on MII maturation rates and fertilization rates. Only one study did not offer cleavage rate (7). Except for one study that underwent frozen embryo blastocyst transfer, other five studies underwent fresh cleavage stage embryo transfers, with varying numbers of embryos transferred (11). Moreover, all six studies gave pregnancy rates but with different definitions of per embryo transfer or per cycle. All six RCTs had FSH priming as the study group and non-FSH stimulated for the control group. Of these studies, three RCTs had additional HCG triggering in the study group, whereas the other three RCTs only had pure FSH priming for their study groups (6, 12, 13). Four RCTs used 150IU FSH for 3 days before retrieval, 1 RCT used 150IU FSH for 2 days before retrieval, and 1 RCT used 75IU FSH for 6 days before retrieval. Embryo transfer was done on D3 in two RCTS, D2–3 in the other three RCTS, and blastocyst transfer in one RCT, whereas the number of embryos transferred varied with each study, ranging from one to two per transfer for blastocyst embryos, and one to six cleavage stage embryos per transfer in different studies.

**Table 1:** Study patient population and study characteristics.

First Author/year (Citation)	Date of ART/Study location	Study design	Study population	Population including PCOS	FSH priminggroup protocol /Control group	Embryo transfer	Primary outcome measures	Relevant outcome measures
Voung LN, 2025 (11)	January 2023 to April2024/ My Duc Hospital in Ho Chi Minh City, Vietnam	Randomized,controlled, assessor-blinded trial	18-37 y/o, infertile with PCOS diagnosis with tubalfactor or male factor/ Exclude: patients undergone IUI or IVF within 3 months, uterine or ovarian abnormal ities, previous low oocyte maturation, or those needing surgicalsperm retrieval	150IU	Intervention: FSH priming 1501U for 2 days / CAPA IVM Control: no priming / CAPA IVM	One good quality blastocyst (3BB or above), or two non- good quality blastocysts / Frozen transfer	Number of matured oocytes, Number of fertilized oocytes, cleavage embryos, blastocysts	Pregnancy rate, Implantation rate, Miscarriage rate,Ongoing pregnancy rate, Live birth rate
Choavaratana R, 2015 (10)	X/ Infertility and Reproductive Biology Unit of Siriraj Hospital, Thailand	Randomized controlled study	18-35 y/o, infertile with PCOS diagnosis/ Exclude: patients with metabolic syndrome, previous ovarian surgery, endometriosis, or those needing surgical sperm retrieval	150IU	Intervention: FSH priming 1501U for 3 days, Ovidrel 250 *µ* g 36 hours before retrieval Control: no priming	2 D3 Embryos transferred / Fresh transfer	Oocyte maturation rate,embryo cleavage rate	Pregnancy and Implantation rates
Fadini R, 2009 (7)	May 2006 to June 2008/ Biogenesi Reproductive Medicine Centre of Monza, Italy	Randomized controlled study	24-38 y/o and normal ovulatory cycles; IVF/ICSI due to male infertility,tuba l factor, stage I/II endometriosis, unexplained infertility Exclude:PCOS, endocr ine abnormalities	150IU	Intervention: FSH priming 1501U for 3 days Control: no priming	1-3 D2 or D3 embryos transferred / Fresh transfer	Oocyte maturation rate,fertilization rate,	Implantation rate, Miscarriage rate
Lin YH, 2003 (9)	Nov 1999 to Dec 2000/ Taiwan	Randomized controlled study	23-39 y/o with PCOS	10,000IU	Intervention: FSH priming 75IU for 6 days,HCG 100001U 36 hours before retrieval Control: HCG 10,000IU 36 hours before retrieval	2-6 D3 embryos transferred / Fresh transfer	Oocyte maturation rate, fertilization rate,cleavage rate	Pregnancy rate, Implantation rate
Mikkelsen AL, 2001 (6)	March 1998 to June 2000 / Denmark	Randomized controlled study	Ages up to 38 y/o,infertility with PCOS diagnosis and with conditions of :tubal damage, male factor infertility, or 3 previous unsuccessfulIVF-ICSIcycles	150IU	Intervention: FSH priming 1501U for 3 days Control: no priming	1-2 D2.5-D3 embryos transferred / Fresh transfer	Oocyte maturation rate, fertilization rate,cleavage rate	Pregnancy rate, Implantation rate
Mikkelsen AL, 2000 (13)	X / Denmark	Randomized controlled study	18-37 y/o,normal ovulatory cycles, Excluded: PCOS, endocrine abnormalities, low ovarian reserve (AFC<3 or FSH >15, inhibit B <45), 3 previous failed IVF cycles	–	Intervention: FSH priming 1501U for 3 days Control: no priming	1-2 D2.5-D3 embryos transferred / Fresh transfer	Oocyte maturation rate,fertilization rate,cleavage rate	Pregnancy rate

### Risk of bias in included studies

**Figure 2:**
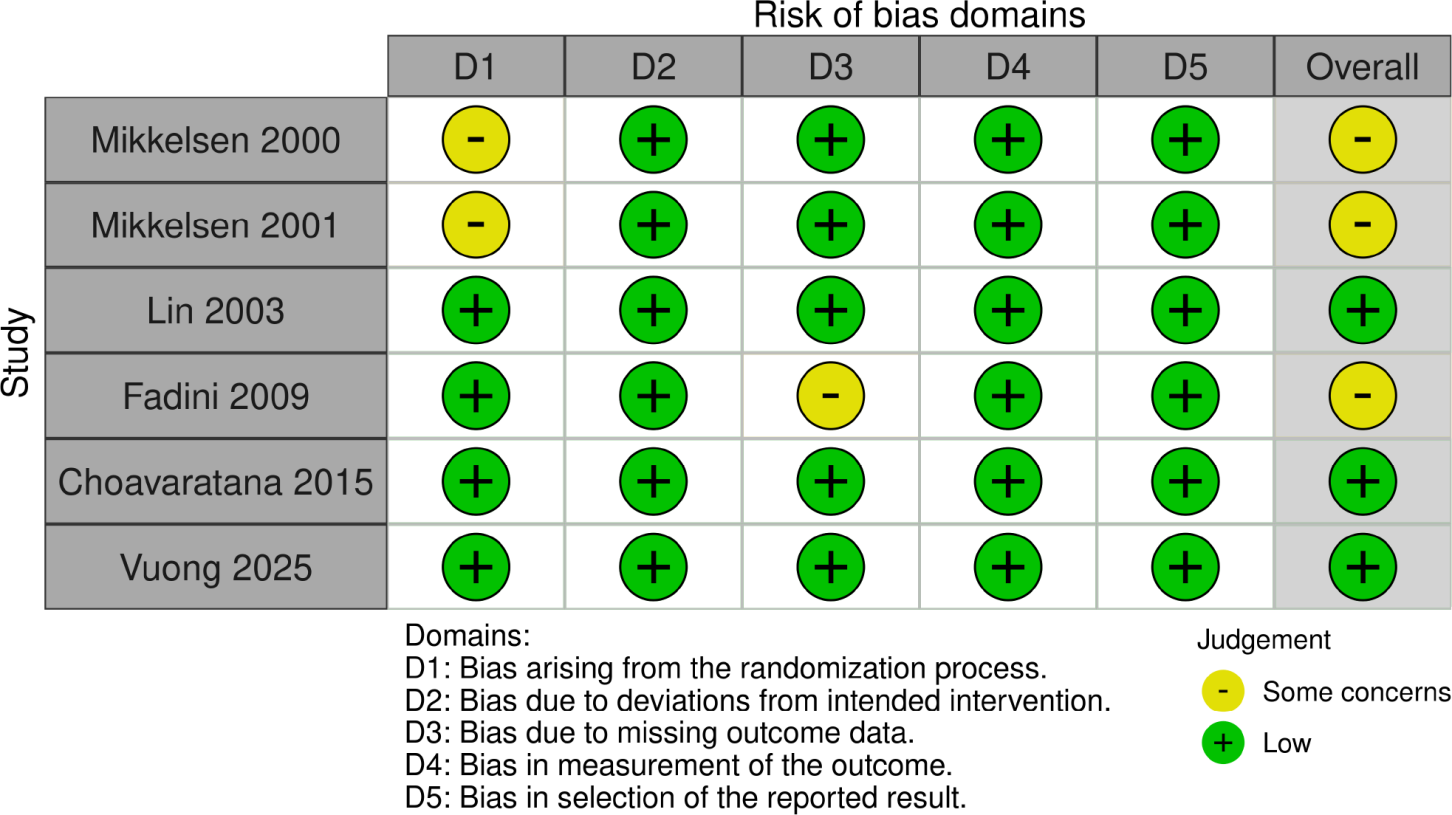
Risk of bias (ROB 2.0).

Risk of bias was assessed for all six included studies, and a traffic-light plot of each domain level for each of the studies is shown in **Figure 2**. Of the six RCTs, four provided clear methods for randomization. These studies used methods of computer-generated randomization listing or block randomization, which was rated as having a low risk of bias (7, 9–11). The remaining two RCTs did not provide detailed information on randomization process and was judged as risks with some concern for the randomization process. With all six RCTs, we considered that even when participants and healthcare providers were aware of their assigned treatment, there were no deviations from intended treatment and were judged as low risk. Five RCTs did not have missing outcome data and were low risk for this domain. One RCT did report suspended participants, some for medical reasons, and some for personal reasons (7). Some concern of risk in this domain was given for this RCT because there was a 6% of missing outcome data, and some reasons include personal reasons for dropout or medical reasons related and/or unrelated to procedure. All six RCTs gave reports without selection on oocyte maturation rates, fertilization rates, and pregnancy outcomes and were assessed as low risks of bias in domain of measurement of outcomes and domain in selection of reported results.

### Primary outcomes

**Figure 3:**
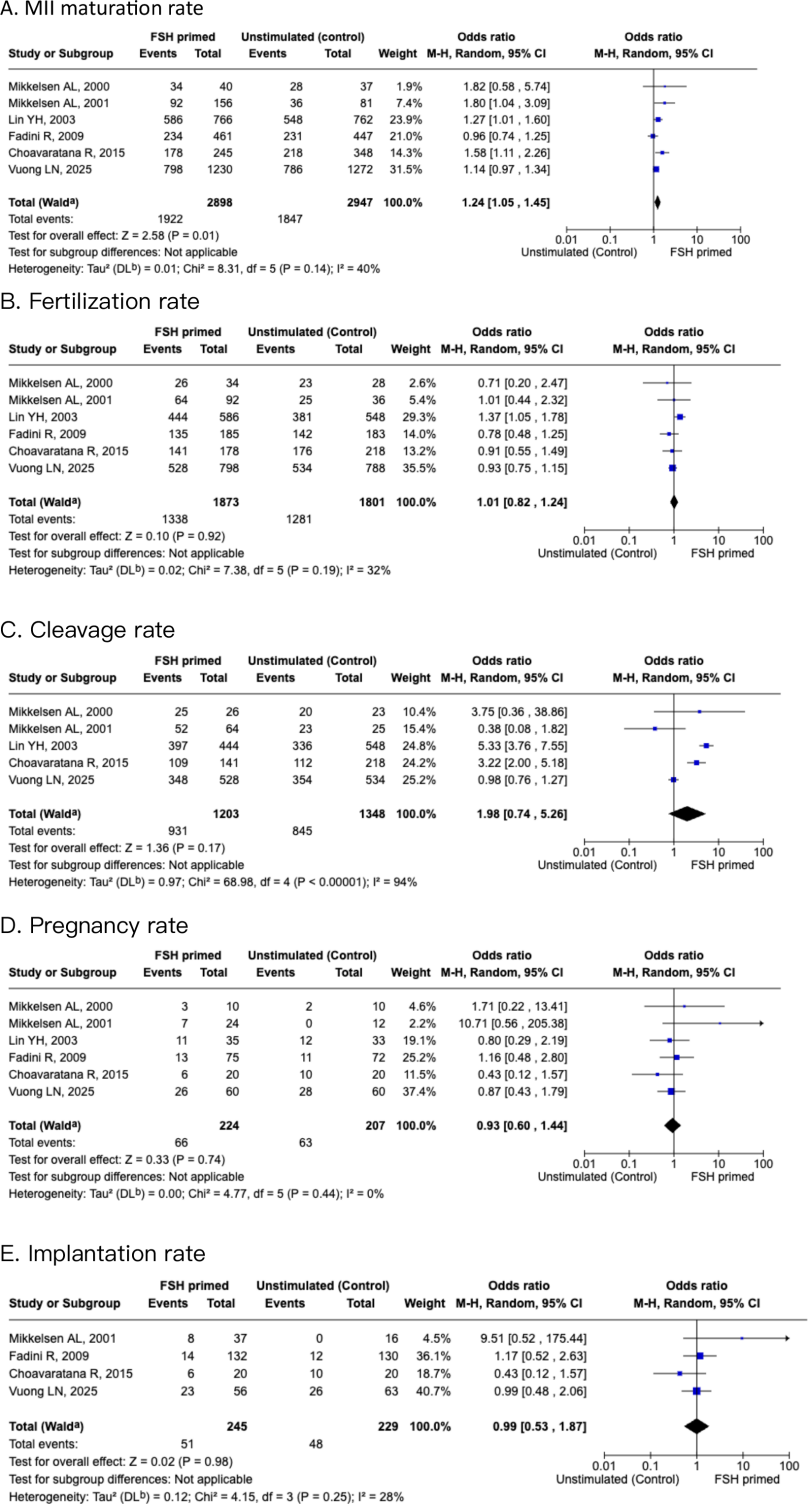
Forest plots of FSH primed and non-stimulated cycles. **(A)** MII maturation rate. **(B)** Fertilization rate. **(C)** Cleavage rate. **(D)** Pregnancy rate. **(E)** Implantation rate.

Of the included six studies in meta-analysis, all studies gave data for oocyte MII maturation rate, defined as percentage of mature oocytes after cumulus-enclosed oocytes were cultured. Meta-analysis showed that the FSH priming group showed significantly better maturation rates than the non-stimulated group [OR 1.24 (95% CI, 1.05-1.45)]. The analysis indicated low heterogeneity (I^2^ = 40%), as shown in **Figure 3**. Fertilization rate and cleavage rate did not show any difference between the FSH-primed group and non-FSH-primed group [OR 1.01 (95% CI, 0.82–1.24), and OR 1.98 (95% CI, 0.74–5.26), respectively], with moderate heterogeneity, I^2^ = 32% and I^2^ = 94%, respectively.

### Secondary outcomes

After the six studies were pooled, the pregnancy rates showed no significant difference [OR 0.93 (95% CI, 0.6-1.44)], with low heterogeneity (I^2^ = 0%). Implantation rates showed no significant difference between the two groups also [OR 0.99 (95% CI, 0.53–1.87)], with moderate heterogeneity (I^2^ = 28%).

### Subgroup analysis of PCOS population

**Figure 4:**
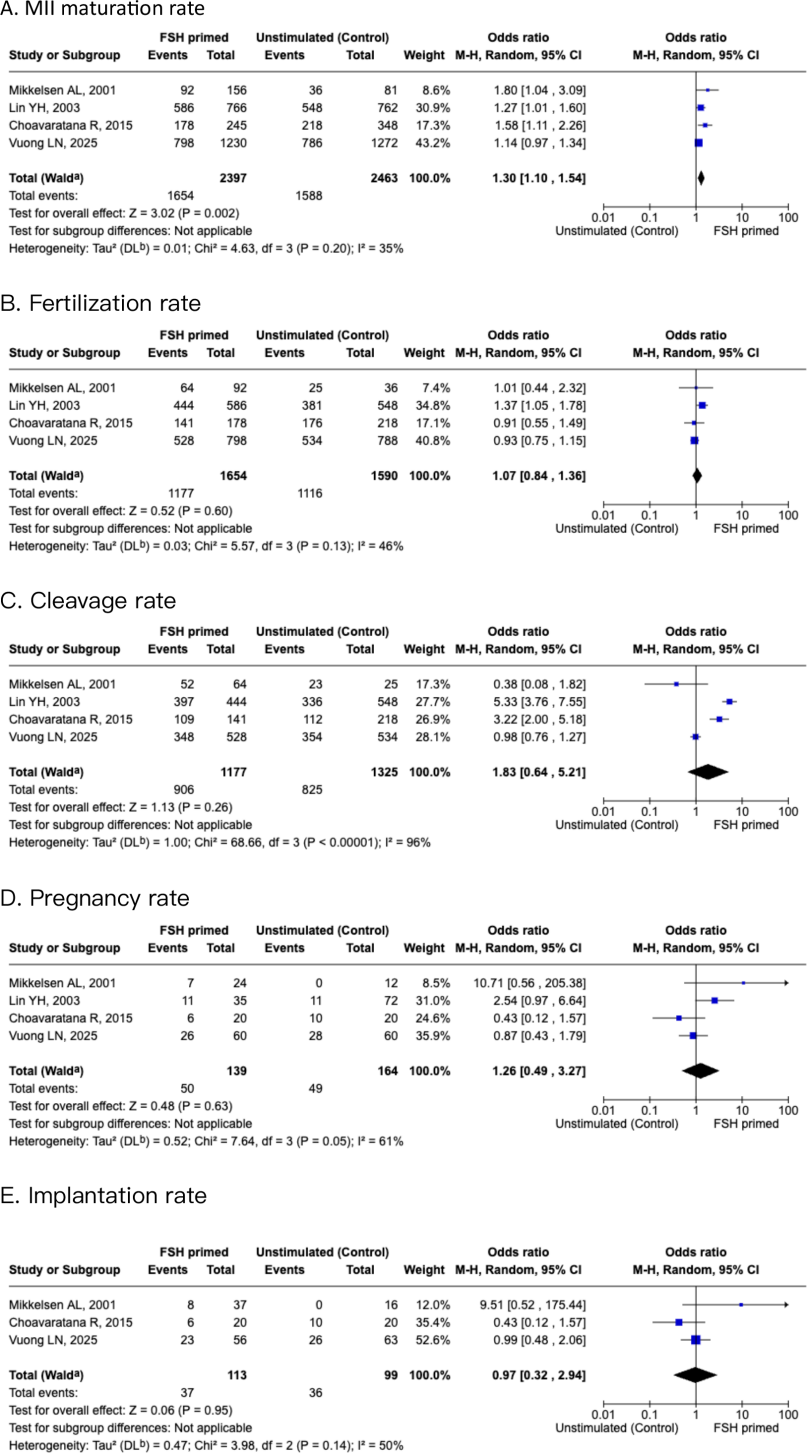
Forest plots of PCOS subgroup of FSH primed and non-stimulated cycles. **(A)** MII maturation rate. **(B)** Fertilization rate. **(C)** Cleavage rate. **(D)** Pregnancy rate. **(E)** Implantation rate.

A subgroup analysis within the IVM cohort examined the population with PCOS for ART outcomes. Four studies compared oocyte MII maturation rate, and fertilization rate, cleavage rate, pregnancy rate, and implantation rate. There was a significant increase in maturation rate of oocytes in the FSH-primed group than the non-stimulated group [OR 1.3 (95% CI, 1.1-1.54, I^2^ = 35%)], as shown in **Figure 4**. There was no significant difference in fertilization rate and cleavage rate for the FSH-primed group when compared with the non-stimulated group [OR 1.07 (95% CI, 0.84–1.36), I^2^ = 46%], and [OR 1.83 (95% CI, 0.64–5.21), I^2^ = 96%], respectively. For pregnancy rate and implantation rate, there were no significant differences within the two groups [OR 1.26 (95% CI, 0.49–3.27), I^2^ = 61%], and [OR 0.97 (95% CI, 0.32–2.94), I^2^ = 50%], respectively.

## Discussion

The impact of FSH priming on oocyte maturation rates in IVM cycles remains a subject of debate. The findings of our meta-analysis demonstrated a significant improvement in MII maturation rates in the FSH-primed group compared with the unstimulated control group (OR 1.24, 95% CI 1.05-1.45, I^2^ = 40%), supporting the notion that FSH exposure enhances nuclear and cytoplasmic maturation (2). This effect is also quite pronounced in PCOS patients, in which our subgroup analysis showed a distinct increase in maturation rates (OR 1.3, 95% CI 1.1-1.54, I^2^ = 35%). FSH mediates cumulus-oocyte communication and upregulation of key maturation-associated genes. FSH stimulation has been shown to promote cumulus expansion and gap junction function, which would facilitate the transfer of essential metabolites to the oocyte and optimizes cytoplasmic competence (14). Mitochondrial activity also plays a strong role in oocyte maturation, with ATP production showing distinct fluctuations during germinal vesicle breakdown (GVBD), spindle migration, and MII transition (15). Studies have shown that mitochondrial clustering and ATP production peaks occur at key maturation checkpoints, emphasizing metabolic regulation that is necessary for meiotic progression (16, 17). The role of granulosa cell-derived factors, for instance, bone morphogenetic protein-15 (BMP-15), or growth differentiation facter-9 (GDF-9), has also been shown to mediate the effects of FSH priming. These factors are secreted by the oocyte and influence cumulus cell function via BMP receptor-mediated SMAD phosphorylation, prompting an optimal follicular environment for maturation (17).

As interesting these findings may be, heterogeneity in study protocols does hinder direct comparison. Variations in FSH dosing regimens (e.g., 150IU for 2 days or 3 days, 75IU for 6 days) may lead to differences in follicular response and oocyte competence. Notably, while some studies demonstrated a positive effect of FSH priming on oocyte maturation rates, there are also some studies finding no significant improvement, suggesting that the benefit of gonadotropin priming may be patient-specific and influenced by additional factors such as patient population and laboratory conditions (2, 14, 18). This underscores previous ongoing debate regarding the necessity and efficacy of FSH priming in IVM protocols. With the results of our meta-analysis, it sheds some light on the benefits of FSH priming with maturation rate, especially on patients with high antral follicle count.

Our meta-analysis did not find a significant difference in fertilization rates between the FSH-primed and non-primed groups (OR 1.01, 95% CI 0.82-1.24, I^2^ = 32%). Nor were there differences in cleavage rates (OR 1.98, 95% CI 0.74-5.26, I^2^ = 94%), indicating that while FSH priming enhances oocyte maturation, it does not necessarily translate to improved early embryonic development. We also did not find a significant difference in pregnancy rates between the FSH-primed and non-primed groups (OR 0.93, 95% CI 0.6-1.44, I^2^ = 0%) or implantation rates (OR 0.99, 95% CI 0.53-1.87, I^2^ = 28%). One study has shown that embryos derived from IVM cycles exhibit higher rates of early developmental arrest, particularly at the two-cell and four-cell stages (19). Walls et al. proposed that this observation is more likely due to incomplete cytoplasmic maturation (19). Additionally, IVM culture conditions, including supplementation with growth factors, or coculture systems, may play an important role in fertilization success and embryo quality. Studies have shown that different culture media can influence oocyte maturation rates. The capacitation IVM (CAPA-IVM) system, which includes a pre-maturation step before the maturation culture, has also been found to enhance oocyte competence and blastocyst development. This demonstrates that the culture media composition plays a critical role in optimizing outcomes (11, 20, 21).

There have been various gonadotropin priming strategies investigated to optimize oocyte maturation in IVM cycles, with studies comparing FSH priming, hCG priming, dual priming (FSH+hCG), and no priming. Some reports propose that combining FSH and hCG priming yields superior maturation and pregnancy rates when compared with FSH or hCG alone, suggesting a potential synergistic effect between these hormones (7, 22). In comparison, one study that compared hCG priming versus no priming has shown that hCG administration before oocyte retrieval leads to faster *in vitro* maturation and improved embryo development (18). However, conflicting results exist, as some studies report that IVM cycles without priming can still achieve reasonable maturation rates, but with a lower clinical pregnancy outcome (23, 24). A recent review by Gotschel et al. (2024) further highlights the ongoing debate, noting that the clinical benefit of FSH priming remains uncertain in fertility preservation cases and thus reflects the need for further well-designed contemporary studies to clarify its role and optimal dosage (5).

Within our meta-analysis, the included RCTs exhibited notable heterogeneity in the patient population, gonadotropin priming regimens, and embryo transfer protocols, which may have potentially influenced the observed differences in outcomes. Regarding the patient population, four studies focused on PCOS patients, whereas the remaining only included normo-ovulatory women with tubal or male factor infertility (6, 9–11). Moreover, differences in FSH dosing regimens and the duration of stimulation were present, with one study employing 75IU of gonadotropins for 6 days and the remaining studies using 150IU of gonadotropins for 2 or 3 days. The impact of additional hCG triggering could potentially complicate comparisons, as three studies utilized hCG triggering, with the rest employing only FSH priming. Embryo transfer strategies differed widely across studies, with two RCTs performing day 3 fresh embryo transfers, and three studies permitted fresh transfers between day 2 and day 3, whereas one utilized frozen blastocyst transfers (9–11). Additionally, the number of embryos transferred varied in all studies, ranging from single to six embryos per transfer, which would heavily confound pregnancy and implantation rates for comparison. These discrepancies in study design elucidates the challenge of drawing collective conclusions on the efficacy of FSH priming in IVM; consequently, this calls for further standardization in patient selection, stimulation protocols, and embryo transfer strategies.

Endometrial receptivity also plays an important role in the success of *in vitro* maturation cycles, having a strong influence on implantation rates and clinical pregnancy outcomes. One of the major concerns with IVM is the potential asynchrony between endometrial development and timing of fresh embryo transfer, as the absence of a substantial endogenous hormones may impair normal endometrial development (22). Furthermore, evidence from freeze-all embryo transfer strategies in IVM cycles has indicated that delayed transfer after endometrial preparation may improve implantation rates (25, 26). Optimizing endometrial receptivity remains a key challenge in IVM cycles, requiring careful synchronization between hormonal support, embryo transfer timing, and luteal phase supplementation. Future research may focus on refining details of endometrial priming protocols and investigating the long-term benefits of freeze-all strategies for IVM cycles to further improve clinical outcomes.

The clinical application of IVM as an alternative to conventional IVF remains a subject of ongoing evaluation, particularly in patient populations at risk of ovarian hyperstimulation syndrome. IVM is associated with significantly lower risks of OHSS, nearly eradicating the risk, which is an appealing option for women with polycystic ovary syndrome and high antral follicle counts (27, 28). While cumulative pregnancy and live birth rates tend to be lower than those observed in conventional IVF, IVM remains a viable alternative in selected patient groups, especially in those who would benefit from avoiding gonadotropin stimulation (28–30). With gained experience and improved technical advancements, evolution in IVM culture systems and protocols continue to improve outcomes, narrowing the gap between IVM and conventional IVF success rates (31). Studies have also highlighted comparable chromosomal integrity in embryos derived from IVM versus conventional IVF cycles, further supporting its feasibility as an alternative ART strategy (32). Furthermore, IVM oocyte retrieval procedures demonstrate safety profiles comparable with conventional IVF, without increased complication rates for cases with high ovarian reserve (33). Another group that may benefit from IVM are cancer patients that need immediate oocyte retrieval or minimal or no stimulation. While this group was not included in the population analyzed in our meta-analysis, IVM may offer advantages for fertility preservation needs (31). While IVM is not yet a direct replacement for conventional IVF, patient selection, protocol optimization, and continued improvements in laboratory techniques will be critical in expanding its clinical utility.

While our meta-analysis provides valuable insights into the role of FSH priming in IVM, several limitations must be considered. Some of the included RCTs had relatively small sample sizes, which could limit statistical power and also the potential to generalize findings across different patient populations. Additionally, heterogeneity in study protocols, e.g., variations in FSH dose, duration and the use of additional hCG priming, and differences in fresh and frozen transfers, complicates direct comparisons between studies. Notably, a recent study Jannaman et al. (2024) compared different FSH priming strategies in PCOS patients and suggested that a minimal 3-day regimen may improve oocyte recovery and developmental potential (34). Given the lack of consensus on the optimal dose and duration of FSH for IVM protocols, these findings highlight the importance of future studies aiming to define and standardize stimulation regimens, as even subtle differences in FSH exposure may impact oocyte quality (35, 36). Another major limitation is the lack of long-term follow-up, as most studies reported implantation and pregnancy rates, and only one gave clear live birth rates. Thus, it remains less clear whether FSH priming has lasting effects on IVM outcomes, including sustained clinical benefits and potential risks. What is noteworthy is that more than half of the RCTs were conducted in the early 2000s, when IVM culture systems, embryo vitrification, and laboratory techniques were less optimized than current standards. With contemporary culture methods and blastocyst formation rates, the outcomes observed in earlier studies may no longer fully represent the potential of IVM technology today. In addition, inconsistencies in endometrial preparation protocols may have influenced implantation success, further limiting the comparability of outcomes.

In light of ongoing efforts to enhance maturation quality and developmental potential in IVM, recent studies on biphasic protocols emphasize the importance of optimizing the hormonal and temporal coordination of oocyte maturation. This is especially evident in the context of fertility preservation, as De Roo et al. (2021) reviewed the application of ovarian tissue oocyte (OTO)-IVM, a method involving *in vitro* maturation of oocytes collected from excised ovarian tissue (36). Within this framework, they highlighted CAPA-IVM, a biphasic protocol that incorporates a pre-maturation culture and C-type natriuretic peptide and EGF-like factors, as a promising approach to improve synchrony between nuclear and cytoplasmic maturation. These advances highlight the growing recognition that modulation of the oocyte environment, whether through priming hormones or sophistications in IVM culture designs, may be essential for improving IVM efficacy and broadening its clinical applications.

As IVM continues to evolve, several key areas warrant further investigation to optimize its clinical potential. While our findings suggest that FSH priming boosts oocyte maturation, especially in PCOS patients, the unanswered question remains: does this advantage translate into better embryo development and pregnancy outcomes? As the field of IVM advances, new evidence will be crucial in determining whether priming strategies can unlock the full potential of IVM. To clarify the impact of FSH priming in IVM, larger and well-designed RCTs using updated culture systems and standardized protocols are needed to improve the robustness of future findings. Additionally, advancements in IVM culture systems are crucial for improving blastocyst development and implantation potential. Emerging strategies such as biphasic IVM (CAPA-IVM), micro-vibration culture, and 3D culture systems also hold promise in mimicking the *in vivo* environment more effectively, potentially enhancing oocyte competence (1). Further research should also focus on long-term follow-up of IVM-derived embryos, addressing concerns related to embryonic development, epigenetic stability, and perinatal outcomes (3). As technological advancements continue to refine IVM protocols, well-powered randomized controlled trials with standardized methodologies will be essential to establish best practices and improve clinical success rates.

## Conclusion

Our systematic review and meta-analysis demonstrated that FSH priming significantly enhances oocyte maturation rates in IVM cycles, particularly in PCOS patients. However, its effects on fertilization, implantation, and clinical pregnancy rates remain inconclusive, with conflicting evidence across studies.The large variability in priming and culture protocols in current studies highlight the need to establish whether FSH priming in IVM truly improves pregnancy and live birth rates. Hence, well-powered, randomized controlled trials are urgently needed. As IVM gains interest as a viable ART alternative, further research should focus on standardizing protocols, optimizing culture conditions, and evaluating long-term safety.

## Data Availability

Publicly available datasets were analyzed in this study. This data can be found here: Data was collected from 6 RCTs, and the direct DOI links are as below: DOI:10.1093/humrep/15.suppl_5.11 DOI:10.1530/rep.0.1220587 DOI:10.1093/humrep/deg335 DOI:10.1016/s1472-6483(10)60168-x DOI:10.1159/000367660 DOI:10.1016/j.fertnstert.2024.09.010.
